# Pharmacokinetics and tolerability of a veterinary phenobarbital product in healthy dogs

**DOI:** 10.3389/fvets.2023.1307888

**Published:** 2024-01-05

**Authors:** Tom Jukier, Amanda Gross, Dawn Boothe

**Affiliations:** ^1^Department of Clinical Sciences, College of Veterinary Medicine, Auburn University, Auburn, AL, United States; ^2^Scott Ritchey Research Center, College of Veterinary Medicine, Auburn University, Auburn, AL, United States; ^3^Department of Anatomy, Physiology, and Pharmacology, College of Veterinary Medicine, Auburn University, Auburn, AL, United States

**Keywords:** phenobarbital, dog, pharmacokinetics, seizure, epilepsy

## Abstract

**Introduction:**

Phenobarbital has been used for many decades in both human and veterinary epileptic patients. Many formulations for a particular drug exist, most of which are marketed for humans. Recently a veterinary specific phenobarbital product has been introduced to the market in the United States. Utilizing a specific formulation to treat patients may help decrease the issue of bioequivalence between one pharmaceutical product to another. Therefore, the goal of this study was to determine single and multiple dosing pharmacokinetics and tolerability of a veterinary specific phenobarbital product over a 4-week time period.

**Materials and methods:**

8 Healthy dogs from a canine research colony were used in the study.

**Results:**

Overall, this phenobarbital formulation was well tolerated in the dogs in this study. Cmax, Tmax, half-life, and AUC after single 12 mg/kg oral dose were 23.5 μg/mL, 4.2 h, 94 h, and 2,758 h*μg/mL. Following chronic dosing, these parameters were 29.1 μg/mL, 3.4 h, 70 h, and 2,971 h*μg/mL, respectively.

**Discussion:**

This formulation demonstrated a mean absolute bioavailability of 100%, with similar pharmacokinetic properties to previously published data.

## 1 Introduction

The use of barbiturates in the dog dates to the early twentieth century, when the first barbiturate drug, diethyl barbituric acid was used to induce sleep in dogs (1). This barbiturate’s effect led to its marketing under the trade name Veronal^®^. Subsequent derivations of this compound lead to the development of phenobarbital in 1911, which was marketed as Luminal^®^. Although it’s intended use was to induce sleep in human patients, phenobarbital’s antiseizure properties were first noted and published in 1912 by serendipitous discovery in a ward of patients with epilepsy (2). By the end of the decade, the first report recommending its use in people with epilepsy was published in 1919 (3). Since then, phenobarbital has been used to help treat epileptic patients in both humans, dogs, cats, and other species.

Phenobarbital has the longest historical use to treat epileptic seizures in dogs (4), and its popularity is still evident based on a survey of neurologists and emergency clinicians in the United States (5). From an efficacy perspective, two consensus reports (4, 6), and a systematic analysis (7) demonstrate evidence for efficacy of phenobarbital, as an antiseizure medication (ASM). Additionally, of the antiseizure medications used in veterinary medicine, only phenobarbital and bromide have established reference intervals to help guide clinicians in making therapeutic decisions (8, 9).

Although there are several previous studies describing the oral pharmacokinetics of phenobarbital in dogs (10–13), none involved approved products (14). Save for a single IV preparation (15), in the United States, no phenobarbital is approved in either humans or dogs because it was among the drugs already on the market when the 1938 Food and Drug Act was enacted, and thus exempt from a new drug status under a grandfather clause (Food and Drug Administration, Compliance Guideline Sec 440 Marketed New Drugs Without Approved NDAs and ANDAs). DailyMed, which in contrast to the Orange Book includes unapproved drugs, lists over 20 phenobarbital products that are unapproved. Any one of these products might be used to treat epileptic seizures in dogs. There is a need for an approved canine product, which would be accompanied by relevant pharmacokinetic information supporting a dosing regimen for the targeted indication.

The purposes of this study were: (1) Describe the pharmacokinetics and oral bioavailability of a new veterinary specific phenobarbital formulation. (2) Describe the pharmacokinetics and oral bioavailability after 4 weeks of once daily therapy at dose designed to maintain therapeutic concentrations; and (3) Assess tolerability after a 4 week dosing period.

## 2 Materials and methods

This study was implemented as a randomized cross-over study using healthy dogs (7 male, 1 female) from a canine colony at the Scott Ritchie Research Center at Auburn University. Health status was determined based on physical examination, neurological examination, complete blood count, and serum biochemistry. The study was conducted in three phases; all dogs were studied in each phase. Phase I consisted of a crossover single 12 mg/kg (or the nearest tablet size for oral administration) dose of phenobarbital administered either orally (NOBATOL^®^ Mizner Bioscience LLC, Boca Raton, FL) to 4 dogs, or intravenously (Hikma Pharmacceuticals PLC, London, United Kingdom, and Cameron Pharmaceuticals, Lousiville, Kentucky) to four dogs. The route of administration was crossed over after a 3-week wash-out period from the last sample collection. A 12 mg/kg dose was calculated to achieve plasma drug concentration (PDC) of 20 μg/mL (4, 8, 16), a concentration considered in the mid therapeutic reference interval, based on the equation Dose = PDC * volume of distribution (16). Phase II commenced immediately after collection of the last sample from phase I. During this time all dogs were treated with 6 mg/kg (to the nearest half tablet size) PO q24h for 4 weeks with dosing occurring in the morning. Complete blood counts and serum biochemistry were obtained for each dog immediately prior to starting this phase, 2 weeks into treatment, and at the end of phase II (week 4). Blood samples for phenobarbital analysis were also collected at 2 (considered peak) and 24 h (considered trough) at the end of weeks, 1, 2, 3, and 4. After the last dose of Phase II, a 7-day wash-out period was allowed during which a blood sample was collected once a day at 24-h intervals. Lastly, phase III commenced on day 7 of the washout period following phase II. Phase III was conducted in a similar manner to phase I at the same mg/kg dose, with the exception that only 7 days elapsed between routes of administration. The funder of this project had no influence on study design.

## 3 Sample collection and handling

One day before blood collection for each phase, jugular catheters (MILA International INC., Florence, Kentucky, United States) were placed in each dog to facilitate blood collection. Dogs were sedated for jugular placement using 375 μg/m2 dexmedetomidine (Dexdomitor^®^ 0.5 mg/mL, Orion Corporation, Espoo, Finland), and if additional sedation was required, 5-10 mg/kg of ketamine (Ketamine hydrochloride injection 100 mg/mL, Covetrus North America, Dublin OH). Dogs were fasted for approximately 14 h prior to phenobarbital administration and remained fasted for 12 h after drug administration. For phase I 3 mL of blood was collected at each time point and placed into a red top tube. For PO administration, samples were collected (hours) at: 0 (prior to drug administration) 0.25, 0.5, 1, 2, 4, 6, 8, 10, 12, 24, 36, 72, 96, 120, 144, and 168. For IV administration, timing was the same with the addition of 0.083 and 0.156 h after dose administration. For phase III, blood samples were at the same time, but only to hour 144. For Phase II, as drug concentrations declined after multiple dosing, during the 7-day washout period, samples were collected at 24, 48, 72, 96, 120, 144, and 168 h. Table 1 shows timeline of the study design. Upon collection, all samples were centrifuged within 2 h at 2500 g for 10 min. Serum was harvested, stored in CRYO.S tubes (greiner BIO ONE, Germany) tubes at −80°C until analysis.

**Table 1:** Representation of dose administered and time points where blood samples were collected.

Phase I* – single 12 mg/kg dose*				Phase II – 6 mg/kg PO q24h	**7 Day washout	Phase III*† − single 12 mg/kg dose			
Dogs 1–4	Administered Orally	28 Day washout	Administered Intravenously	‡28 Day administration		Dogs 1–4	Administered Orally	7 Day washout	†Administered Intravenously
Dogs 5–8	Administered Intravenously		Administered Orally			Dogs 5–8	Administered Intravenously		†Administered Orally

*PO timepoints: 0, 0.25, 0.5, 1, 2, 4, 6, 8, 10, 12, 24, 36, 72, 96, 120, 144, and 168. *IV timepoints: 0, 0.08, 0.16, 0.25, 0.5, 1, 2, 4, 6, 8, 10, 12, 24, 36, 72, 96, 120, 144, and 168. †Four dogs had blood samples collected up to (and including) hour 144. ‡Peak and trough concentrations were collected at days 7, 14, 21, and 28. **Timepoints 24, 48, 72, 96, 120, 144, and 168 h.

Serum phenobarbital was quantitated using a Siemens (Malvern, PA) Dimension^®^ PHNO homogenous particle enhanced turbidimetric inhibition immunoassay (PETINIA) (10444933), carried out on a Siemens Dimension^®^ Xpand^®^ Plus integrated clinical chemistry system. This assay is approved by the US Food and Drug Administration for use in human medicine. The assay is calibrated using the Siemens Dimension^®^ DRUG CAL (10445014) serum calibrator set with concentrations ranging from 0-80 μg/mL. The lower limit of quantitation is 1 mcg/ml and the CV is 4.42% at 14 μg/mL in pooled serum and 4.72% at 28.4 μg/mL in pooled plasma (*n* = 40).

## 4 Pharmacokinetic and statistical analysis

Serum phenobarbital concentrations versus time curves were analyzed by non-compartmental analysis using pharmacokinetic software (Phoenix WinNonLin^®^, CERTARA, United States). Non-compartmental analysis was performed using the linear-log up down trapezoidal option for determination of the area under the time-concentration curve (AUC) versus time (Phoenix WinNonlin^®^). From this, the following were determined: mean residence time (MRT), disappearance rate constant (k_el_), terminal half-life (t_1/2_, determined from the relationship t_1/2_ = 0.693/k_el_), area under the curve to infinity (AUC_∞_), percent of the AUC that was extrapolated from the terminal component of the curve (AUC ext), and, in the absence of IV administration (the decline of phenbobarbital concentrations during Phase II), the apparent volume of distribution (Vz) and clearance (Cl). Maximum plasma drug concentration (C_max_), and time to maximum concentration (T_max_) were observed. Data was reported both as a mean and standard deviation (SD) and as median and range (minimum and maximum values) with the exception of half-life, which was reported as harmonic mean ± pseudostandard deviation. Absolute bioavailability was calculated based on (AUC_oral_*DOSE_IV_/AUC_IV_*DOSE_oral_) × 100.

Kolmogorov–Smirnov test was performed to assess normality of the data. Comparison of oral Tmax, Cmax, absolute bioavailability, terminal half-life, were compared using paired student t-test or Mann–Whitney test, while blood work parameters were compared using One-Way Analysis of Variance (ANOVA) or Friedman’s test was used to assess parameters from all groups, followed by multiple comparison (Tukey’s for parametric data, Dunn’s test for nonparametric data). Bonferroni correction was applied when assessing for statistical significance within blood work parameters. Statistical significance was set at an α <0.05. Statistical analysis was performed using GraphPad Prism 9.5.1 (Dotmatics, San Diego, CA).

## 5 Results

Mean ± SD of dog weights included in the study was 13.4 ± 3.2, and mean age was 6.0 ± 2.9 years. Table 2 shows demographic information about each dog in this study. Mean phenobarbital dose was as follows: Phase I PO 11.9 ± 0.1, phase I IV 12.1 ± 0.4, phase II 6.2 ± 0.4 q24h, phase III PO 12.2 ± 0.5, and phase III IV 12.3 ± 0.4 mg/kg.

**Table 2:** Demographics of dogs in the study.

Animal	Weight (Kg)	Age (Years)	Sex	Breed
1	15.1	5.1	MI	Corgi beagle cross
2	12.5	5.2	MI	Beagle
3	15.2	5.1	FI	Corgi beagle cross
4	15	11	MI	Corgi beagle cross
5	14.2	10	MI	Labradoodle
6	8	3.2	MI	Beagle
7	9.3	3.2	MI	Beagle
8	17.6	5.1	MI	Corgi beagle cross

### 5.1 Phase I vs. phase III – single dose pharmacokinetics across time

**Figure 1:**
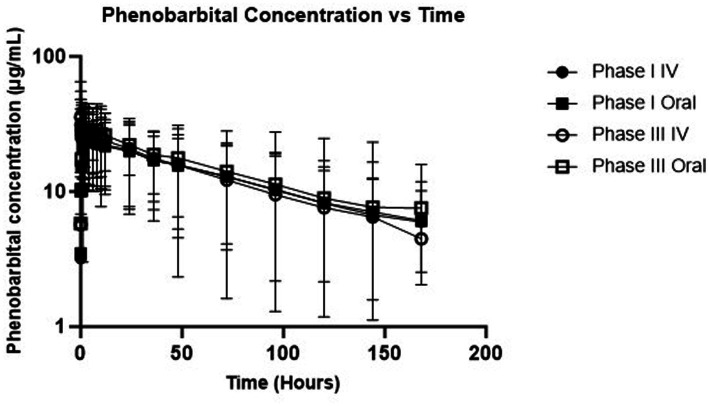
Semi-logarithmic depiction of phenobarbital concentration (with standard deviation bars) vs. time for single dose administration (phases I and III).

Descriptive statistics for pharmacokinetic parameters and oral bioavailability are presented in Table 3. Administration of the calculated loading dose yielded a C_max_ of 23.5 ± 3.2 μg/mL at phase I which was lower than that achieved after phase III, 29.1 ± 4.1 μg/mL (*p* = 0.0002). The T_max_ (hr) of phase I was 4.2 ± 2.7 compared to 3.4 ± 1.9 for Phase III (*p* = 0.45). For IV administration, the C_0_ after Phase I was 17.3 ± 2.7 μg/mL compared to 20.6 ± 1.9 μg/mL for phase III (*p* = 0.02). Clearance for IV administration of phase I (6.2 ± 1.5 mL/kg/h) and phase III (6.3 ± 1.3 mL/kg/h) was not statistically different (*p* = 0.81). Mean absolute bioavailability (F) was 1.0 ± 0.1 for Phase I and 1.1 ± 0.1 for phase III. Figure 1 shows mean ± SD phenobarbital concentration vs. time for all dogs.

**Table 3:** Pharmacokinetic parameters for single, multiple, and single-post multiple dosing, of the veterinary specific phenobarbital product used in this study (Table 1).

	Phase I		Phase II	Phase III	
	Oral	IV	Oral	Oral	IV
Dose mg/kg mean ± SD	11.9 ± 0.1	12.1 ± 0.4	6.2 ± 0.4	12.2 ± 0.5	12.3 ± 0.4
Dosing schedule	Single	Single	Multiple	Single	Single
C_0_/Cmax (μg/mL)*	23.5 ± 3.2	17.2	NA	29.1 ± 4.1	20.6
Tmax (hours)	4.2 ± 2.7	NA	NA	3.4 ± 1.9	NA
Terminal half-life (hours)**	80	81.2	94	70	67
AUC_0 → ∞_ (h*μg/mL)	2,758 ± 544	2,751 ± 676	2,614 ± 866	2,971 ± 986	2,661 ± 827
Vz (L/kg)	NA	0.7 ± 0.1	NA	NA	0.6 ± 0.1
Vss (L/kg)	NA	0.7 ± 0.1	NA	NA	0.6 ± 0.06
Cl (mL/h/kg)	NA	6.2 ± 1.5	NA	NA	6.3 ± 1.3
F	1 ± 0.1	NA	NA	1.1 ± 0.1	NA

Mean ± SD for the main pharmacokinetic parameters calculated for each phase of the study. *Value represents that for C_0_ intravenous routes, and C_max_ for oral routes. **Half-life is presented as a harmonic mean. AUC – Area under time concentration curve. CL-Clearance. C_0_ – Initial concentration. C_max_ – maximal concentration. T_max_ – time to maximal concentration. Vz – Apparent volume of distribution of the central compartment. Vss – Apparent volume of distribution at steady state. F – bioavailability.

### 5.2 Phase II – multiple dosing

Specific pharmacokinetic parameters for this phase of the study are presented in Table 3. Weekly mean ± SD phenobarbital concentrations and percentage fluctuation during the 24-h dosing interval are presented in Table 4. Approximately 25% fluctuation in plasma drug concentrations occurred over the 24-h dosing interval.

**Table 4:** Mean ± SD peak and trough concentrations for dogs during the Phase II daily administration of oral phenobarbital.

Week	Peak (μg/mL)	Trough (μg/mL)	% Fluctuation
1	36.3 ± 4.3	26.8 ± 3.2	26.0 ± 8.3
2	29.4 ± 2.7	22.2 ± 3.1	24.5 ± 8.4
3	33.0 ± 6.4	23.5 ± 3.3	28.0 ± 5.8
4	28.2 ± 4.5	20.2 ± 4.7	26.8 ± 27.5*

Trough samples are collected 24 h after dosing. *Dog 5 had a higher trough concentration when compared to peak concentration (20.3 μg/mL vs. 28.3 μg/mL).

### 5.3 Serum biochemical analysis and complete blood counts

During the study period, dogs there were seldom notes about singular episodes of diarrhea. More frequently, slower or no interest in food in the mornings was noted. However, comparison of weight prior to the onset of the study and at the end did not reveal as statistically significant difference. Descriptive statistics including statistical comparisons are presented in Table 5 (serum biochemistry) and Table 6 (completed blood count). Both ALP and ALT remained within the reference interval during the 4-week daily administration. However, a statistically significant difference (*p* = 0.0014) was found between medians between the pre-ALP (36 IU, range 17–136) and median post ALP (81.5 IU/L, range 36–258). A statistically significant difference could not be found in when comparing medians for ALT (*p* = 0.07). All means at each time point were all within laboratory reference intervals with the exception for glucose at week 4. Mean glucose concentrations for the 8 dogs was below reference values. Hypoglycemia was ruled out using an in-house glucose test that confirmed euglycemia. For each dog with a hypoglycemic result, an in-house glucose test was performed to ensure euglycemia.

**Table 5:** Serum biochemistry results (and reference intervals from the laboratory) from dogs prior to 4-week administration (pre), 2 weeks into administration (mid), and at the termination of 4 weeks of administration (post).

Parameter	Reference interval	Pre	Mid	Post	*p*-value
Total protein	5.0–7.4 g/dL	5.4 ± 0.4	5.6 ± 0.2	5.5 ± 0.4	0.33
Albumin	2.7–4.4 g/dL	3.0 ± 0.4	3.0 ± 0.2	2.9 ± 0.3	0.51
Globulin	1.6–3.6 g/dL	2.5 ± 0.2	2.6 ± 0.3	2.6 ± 0.5	0.54
Albumin:Globulin	0.8–2.0	1.3 ± 0.2	1.2 ± 0.2	1.2 ± 0.3	0.85
AST	15–66 IU/L	15.6 ± 1.7	18.0 ± 3.5	22.0 ± 3.8*	0.0018
ALT	12–118 IU/L	26.6 ± 6.4	36.1 ± 16.7	32.4 ± 6.5	0.069
ALP	5–131 IU/L	36.0 (17.0–136.0)	68.0 (30.0–245.0)	81.5 (36.0–258.0)**	0.0009
GGT	1–12 IU/L	3.3 ± 1.0	4.9 ± 1.1	5.4 ± 2.7	0.0095†
Tbili	0.1–0.3 mg/dL	0.1 (0.1–0.1)	0.1 (0.1–0.2)	0.1 (0.1–0.2)	0.33
BUN	6–31 mg/dL	13.8 ± 4.7	13.6 ± 2.7	13.4 ± 3.2	0.88
Creatinine	0.5–1.6 mg/dL	0.7 ± 0.2	0.8 ± 0.2	0.8 ± 0.2	0.1
BUN:Cre	4–27	18.5 ± 1.2	18.6 ± 5.6	16.9 ± 4.6	0.22
Phosphorous	2.5–6.0 mg/dL	3.9 ± 1.4	3.4 ± 0.3	3.7 ± 0.4	0.7
Glucose	70–138 mg/dL	78.6 ± 11.3	79.1 ± 6.9	54.6 ± 10‡	<0.0001
Calcium	8.9–11.4 mg/dL	9.2 ± 0.7	9.4 ± 0.6	9.7 ± 0.4	0.1
Corrected Calcium	NA	9.7 ± 0.4	9.8 ± 0.6	10.3 ± 0.5	NA
Magnesium	1.5–2.5 mEq/L	1.8 ± 0.1	1.3 ± 0.1	1.6 ± 0.1*	0.003
Sodium	139–154 mEq/L	149.1 ± 1.7	148.4 ± 2.3	148.3 ± 1.8	0.63
Potassium	3.6–5.5 mEq/L	4.2 ± 0.4	4.6 ± 0.2	4.9 ± 0.3	0.0002***
Na:K	27–38	35.6 ± 3.0	32.4 ± 2.0	30.5 ± 1.9**	<0.0001
Chloride	102–120 mEq/L	113.3 ± 2.0	111.0 ± 2.6**	109.3 ± 2.5**	0.023
Cholesterol	94–324 mg/dL	179 (116–409)	205.5 (170–741)**	184.5 (124–628)	0.01
Triglycerides	29–291 mg/dL	54.5 (41–337)	57 (27–1,068)	48 (26–751)	0.12
Amylase	290–1,125 IU/L	669.8 ± 246.5	583.8 ± 121.0	628.3 ± 179.3	0.65
Precision PSL	24–140 U/L	79.3 ± 113.5	40.3 ± 21.1	59.6 ± 53.2	0.32
Creatine Kinase	59–895 IU/L	63.6 ± 19.9	95.0 ± 89.1	119.1 ± 52.3**	0.005

For each parameter, if all groups are normally distributed, data is presented as mean ± SD, if all groups were not normally distribution data is presented as Median (Range). *Statistically significantly different than the other two groups. **Statistically significantly different compared to pre timepoint. ***All means statistically significant from one another. †No statistically significant difference found after multiple comparisons. Descriptive statistics of complete blood counts during multiple dosing phase (phase II).

**Table 6:** Mean ± SD of selected complete blood counts results (and reference intervals from the laboratory) from dogs prior to 4-week administration (pre), 2 weeks into administration (mid), and at the termination of 4 weeks of administration (post).

Parameter	Reference interval	Pre	Mid	Post	*p*-value
WBC	4.0–15.5×10^3^/μL	8.738 ± 0.9841	8.65 ± 0.9274	7.813 ± 0.7846**	0.01
RBC	4.8–9.3×10^3^/μL	6.725 ± 0.377	6.888 ± 0.4824	6.825 ± 0.4803	0.53
HCT	36–60%	48.88 ± 2.588	49.88 ± 2.9	51 ± 3.742	0.3
Platelet	170-400×10^3^/μL	341.8 ± 124.5	411.4 ± 121.8	346.3 ± 128.4	0.04†
Neutrophils	2060–10,600/μL	5,609 ± 1,253	5,390 ± 823.1	4,729 ± 792.6	0.068
Lymphocytes	690–4,500/μL	2,288 ± 791.5	2,291 ± 689.8	2094 ± 693.7	0.45
Monocytes	0–840/μL	370 ± 80.04	400.1 ± 121.7	377.8 ± 98.91	0.5
Eosinophils	0–1,200/μL	470.4 ± 218.1	568.1 ± 219	612.3 ± 232.2	0.069

All data is expressed as mean ± SD.

## 6 Discussion

Several decades have elapsed since the pharmacokinetics of phenobarbital have been described (10–13, 17, 18). Table 7 shows a summary of previously reported pharmacokinetic parameters for phenobarbital in dogs. This study demonstrates that the veterinary specific phenobarbital product shows excellent bioavailability (F) in dogs with near complete absorption through the oral route of administration, and at 6 mg/kg/d drug concentrations within the reference interval should be achieved. For intravenous administration, the apparent volume of distribution of the central compartment (Vz) and the apparent volume of distribution at steady-state (Vss) for phase III was 0.6 ± 0.1 L/kg and 0.6 ± 0.06 L/kg compared to 0.7 ± 1 and 0.7 ± 0.1 L/kg for phase I, and difference found to be statistically significantly different (*p* = 0.04), which may account for the higher C_max_ seen during phase III of the study. This appears to be the case given that weights did not differ prior to the study and at completion, and that AUC were similar.

**Table 7:** Previously reported pharmacokinetic data in dogs.

Author	Frey et al. (17)		Ravis et al. (10)	Al-Tahan et al. (18)		Pedersoli (12)				Thurman et al. (13)		Ravis et al. (11)				Bankstahl et al. (37)	
	Beagles	Mongrel dogs	5 Day duration			Intravenous routes		Oral routes		Day 1	Day 22	Day 1 (single dose)	Day 90 (multiple dose)	Day 1 (single dose)	Day 90 (multiple dose)	Luminal ^®^	Phenoleptil ^®^
Dose administered	10 mg/kg	10 mg/kg	6 mg/kg/d	10 mg/kg	10 mg/kg	5.5 mg/kg	15 mg/kg	5.5 mg/kg	15 mg/kg	5 mg/kg/d	5 mg/kg/d	5.5 mg/kg	5.5 mg/kg/d	15 mg/kg/d	15 mg/kg/d	100/dog	100/dog
Route	IV	IV	PO	IV	PO	IV	IV	PO	PO	PO	PO	PO	PO	PO	PO	PO	PO
C_0_/Cmax (μg/mL)	17 ± 1.0	21 ± 2.1	13.2–24.2	21 (13–33)	15 (14–17)	‡	‡	‡	‡	§28.0 ± 2.8	§80.5 ± 6.6	8.9 ± 1.7	27.7 ± 4.0	22.2 ± 2.8	40.5 ± 4.1	10.9 ± 0.92	10.5 ± 0.77
Tmax (hours)	NA	NA	3.2 ± 1.0	NA	6 (4–8)	‡	‡	‡	‡	2	44,961	3.8 ± 1.5	4.6 ± 0.9	4.4 ± 1.5	2.8 ± 1.5	3.75 ± 0.79	2.75 ± 0.37
Disappearance half-life (hours)	32 ± 4.8	10 ± 16	53 ± 15	56 (42–72)	52 (44–62)	92.6 ± 23.7	72.3 ± 15.5	‡	‡	46.3 ± 11.3	29.3 ± 4.6	88.7 ± 19.6	47.3 ± 10.7	99.6 ± 22.6	31.1 ± 4.4	50.3 ± 3.1	52.9 ± 2.8
AUC0 → ∞**	‡	‡	‡	‡	‡	‡	‡	‡	‡	1659.2 ± 186.5	1493.1 ± 205.4	‡	‡	‡	‡	807 ± 66.8	781 ± 54.0
Vd (L/kg)	0.6 ± 0.04	0.68 ± 0.03	0.74 ± 0.21*	‡	‡	0.7 ± 0.15	0.69 ± 0.14	‡	‡	‡	‡	706 ± 129*	720 ± 175*	736 ± 107	712 ± 144	‡	‡
Cl (mL/h/kg)	13 ± 1.7	7.0 ± 1.3	10.4 ± 3.2†	‡	‡	5.6 ± 2.3	6.7 ± 0.8	‡	‡	‡	13.3 ± 1.3†	5.6 ± 1.9†	10.2 ± 1.7†	7.3 ± 1.1	15.6 ± 2.5	‡	‡
F	NA	NA	NA		91 (86–96)	NA	NA	0.88 ± 0.12	0.97 ± 0.09	‡	‡	‡	‡	‡	‡	0.98 ± 0.031	

C_0_ – extrapolated maximal concentrations for intravenous administration. C_max_ – maximal concentration for oral administration. T_max_ – Time to maximal concentration. Vd – apparent volume of distribution. Cl – Clearance. F – Bioavailability. *Value represents Vd uncorrected for bioavailability (Vd/F). **Units for AUC published as μmol/h/l. †Value represents clearance with uncorrected for bioavailability (CL/F). §Authors report in μmol/L; conversion to μg/mL leads to Day 1 6.5 ± 0.65, and Day 22 19 ± 1.5. ‡Not reported.

Mean harmonic half-lives were not statistically different between the phases and routes and ranged from 67–94 h. When assessing peak and trough concentration between each week, approximately 25% fluctuation in PDC occurred during a 24-dosing interval. This suggests that a q24 hour dosing interval may be insufficient for phenobarbital given its longer half-life, and that a q12h dosing interval is justified.

We calculated a loading dose of ~12 mg/kg for phase I, single dose studies based on a previously reported Vd of 0.7 L/kg for dogs and targeting a PDC of 20 μg/mL. Our loading dose appears to have achieved the concentrations within the reported reference interval of 15-45 μg/mL (8) for both IV and PO administration. Furthermore, the maintenance dose used here (6 mg/kg/d) maintained concentrations within the reference interval, and a 3 mg/kg dose q12h may serve as a good starting point for a patient with epilepsy. As such, this dose could be used when a clinician is aiming to achieve a concentration that should produce a therapeutic effect in a patient that requires a useful concentration more immediately. Having said that, although the reference interval is 15-45 μg/mL, concentrations >35 μg/mL may be associated with an increased risk of hepatotoxicity (4, 19). When this dose was administered to the dogs in this study, minimal side effects were noted. However, it would be important to acknowledge that none of these patients had epileptic seizures, a patient that has experienced recent epileptic seizures, or a patient with a structural cause for epilepsy may be more prone to display an adverse effect from such high doses given over a short period of time.

Despite reports of autoinduction associated with phenobarbital induction of cytochrome 450 metabolism, we were not able to demonstrate an expected increased clearance or shortened elimination half-life of phenobarbital over the 4-week treatment period. Indeed, clearance was decreased at 4-weeks compared to baseline. It has been previously demonstrated that clearance was higher in dogs receiving a higher phenobarbital dose (escalating doses starting at ~1.2 mg/kg/d and ending at 32 mg/kg/d) (20), and in dogs treated with phenobarbital over a longer period of time (11). In the latter study, a significantly higher clearance was found 30, 60, and 90 days from initial phenobarbital therapy when compared to the single dose. Furthermore, in a study investigating the lasting effects of liver enzyme induction from a 10 mg/kg/d dose of phenobarbital after a 34 day administration, activity remained enhanced for 4 weeks after cessation of phenobarbital administration in dogs administered antipyrine (21). In this study, initial clearance of 6.2 ± 1.5 mL/kg/h was not statistically different (*p* = 0.8) from that at the end of the study at 6.3 ± 1.3 mL/kg/h It is possible that dogs in this study were not treated with phenobarbital long enough to demonstrate an increase in clearance or there may be individual variation in autoinduction.

Overall phenobarbital was well tolerated in all dogs in both forms. Slight ataxia was noted after the intravenous administration in a couple of dogs, but no dog was profoundly sedated. On blood work evaluation, all parameters except for glucose concentrations remained within laboratory reference intervals. The 4-week glucose concentrations had a mean consistent with hypoglycemia. Since an outside laboratory (ANTECH^®^, Antech diagnostics, Inc) was used for this diagnostic, the hypoglycemia most likely represents improper sample handling and processing prior to submission into the lab, rather than true hypoglycemia. In all dogs that had a hypoglycemic value at any time point, an in-house glucose test was performed as soon as possible to ensure that the laboratory value was spurious. Liver enzymes remained within laboratory reference intervals at each time point. However, an increase in serum ALP, and AST was noted at the 4-week mark when compared to the pre-and mid-point values. The increases in ALP have previously been demonstrated to be largely from induction of corticosteroid induced alkaline phosphatase activity (22, 23).

There are several reports assessing bone marrow suppression in relation to the use of phenobarbital (24, 25). All CBC parameters remained within laboratory reference intervals throughout the 4-week dosing period. The only statistically significant difference found involved total white blood cell counts, with the 4-week time point being lower than the mid-point (*p* = 0.01).

Of important note for therapeutics is the individual patient. This study did not address the potential role of pharmacogenomics and its role in variable phenobarbital pharmacokinetics. Phenobarbital has been demonstrated to induce multiple types for CYP enzymes including CYP1A, CYP2B, CYP2C, and CYP3A (26). With evidence for polymorphisms in various CYP enzymes in the dog (27–35), it is reasonable to think that this may be important when considering therapeutic outcome (or development of adverse effects). As such, therapeutic drug monitoring might serve as a powerful tool to help guide the clinician when making decisions regarding dose and dosing interval. An example involving phenobarbital therapy in dogs demonstrated improved control of epileptic seizures in dogs switching to a from a q12h to q8h dosing schedule (36).

A study assessing bioequivalence between two European approved phenobarbital products has been done (37). This study determined that the two products showed similar relative bioavailability and pharmacokinetic parameters including C_max_, T_max_, elimination half-life, and AUC. This suggests that the two specific products used are bioequivalent to eachother, which is important for therapeutic success, and preventing toxicity. Direct comparison between pharmacokinetic data cannot be made as a set dose per dog of 100 mg was administered. This would affect AUC and Cmax values obtained. Having said that, calculated elimination half-life and T_max_ were more closely aligned between the two studies.

Other limitations of the study included a lack of female dogs in the study (7 males to 1 female). Pharmacokinetics and adverse drug reactions can be different between males and females (38). Although from an ASM efficacy perspective, no definitive differences exist between sexes (39).

Lastly, during the writing of this manuscript a different veterinary specific phenobarbital product was given conditional approval from the FDA. However, to the author’s knowledge, this is the only veterinary specific phenobarbital product with pharmacokinetic data in dogs.

## 7 Conclusion

The veterinary specific phenobarbital formulation to be marketed as NOBATOL^®^ shows near complete systemic absorption, and pharmacokinetic parameters similar to those previously published. The formulation appears to be safe in the dog, the utilization of a consistent formulation may allow for better dose titrations and prevent variations in plasma drug concentration in relation to when one generic product is changed to another within individual patients.

## Data availability statement

The raw data supporting the conclusions of this article will be made available by the authors, without undue reservation.

## Ethics statement

The animal study was approved by Animal Care and Use, Auburn University. The study was conducted in accordance with the local legislation and institutional requirements.

## Author contributions

TJ: Conceptualization, Data curation, Formal analysis, Funding acquisition, Investigation, Methodology, Project administration, Supervision, Writing – original draft, Writing – review & editing. AG: Data curation, Project administration, Resources, Supervision, Writing – review & editing. DB: Conceptualization, Formal analysis, Investigation, Methodology, Resources, Writing – review & editing.
